# Culture Medium for Gonococcus

**Published:** 1914-02

**Authors:** 


CULTURE MEDIUM FOR GONOCOCCUS. A. Lumiere and J. Chrevotier, *Prog. Med.*, December 20, 1913, 6 grams of albumin in looo c·c. of beer must is sterilized at 115. C., filtered hot and neutralized; again sterilized at 110 for ten minutes. As used, it is advantageous but not necessary to add 10% of horse or ass serum. Cultures may be made in 8 hours.

